# Amyotrophic Lateral Sclerosis: Pathophysiological Mechanisms and Treatment Strategies (Part 2)

**DOI:** 10.3390/ijms26115240

**Published:** 2025-05-29

**Authors:** Christina Tolochko, Olga Shiryaeva, Tatiana Alekseeva, Vyacheslav Dyachuk

**Affiliations:** 1 V. A. Almazov Federal National Medical Research Centre, Saint Petersburg 197341, Russia; tolochko_ka@almazovcentre.ru; 2 Research Laboratory of Neurogenesis and Neurodegenerative Diseases, V. A. Almazov Federal National Medical Research Centre, Saint Petersburg 197341, Russia; olya.shiryaeva.01@mail.ru

**Keywords:** motor neuron disease, amyotrophic lateral sclerosis, pathogenesis, pathogenetic therapy, riluzole, edaravone

## Abstract

Amyotrophic lateral sclerosis (ALS) is a progressive neurodegenerative disease associated with damage to motor neurons and leading to severe muscle weakness and, eventually, death. Over the past decade, understanding of the key pathogenetic links of ALS, including glutamate-mediated excitotoxicity and oxidative stress, has significantly advanced. This review considers the recent evidence on molecular mechanisms of these processes, as well as the therapeutic strategies aimed at their modulation. Special attention is paid to antiglutamatergic and antioxidant drugs as approaches to the ALS pathogenetic therapy.

## 1. Introduction

Amyotrophic lateral sclerosis (ALS) is a fatal progressive neurodegenerative disorder characterized by selective death of motor neurons of the brain and spinal cord, manifested as increasing weakness of skeletal muscles of the limbs, trunk, and respiratory and bulbar muscles, severe motor dysfunction, and respiratory failure, leading to death 2–5 years after the onset of the first symptoms [1].

The disease most commonly begins at an older age, usually in 58–63-yr-olds [2,3]. In rarer cases, ALS appears at a younger (under 40 yr) or juvenile age (under 25 yr) [4,5,6]. The total risk of the disease is higher in men than in women (1:350 vs. 1:400) [7,8].

In terms of incidence, ALS ranks third among neurodegenerative diseases after Alzheimer’s and Parkinson’s diseases [9]. About two new cases of ALS per 100,000 population are registered annually worldwide [2]. The total global incidence of ALS is 4.42 per 100,000 person-years, but this parameter ranges widely from 1.57 per 100,000 person-years in Iran to 10–12 per 100,000 person-years in Europe and 11.80 per 100,000 person-years in the United States [10].

Of particular interest is the steady increase in ALS incidence rates in recent years, especially in countries with developed healthcare systems. Specifically, in Italy, an increase in ALS prevalence was observed from 2015 to 2019, rising from 15.26 per 100,000 to 18.31 per 100,000 individuals in the population [11]. According to the forecasts by Arthur et al. [12], the number of patients is expected to increase by about 20–30% by 2040. This growth is projected to be most pronounced in developing countries, such as Iran, Libya, Serbia, and Uruguay, where ALS cases are predicted to increase by 50% between 2015 and 2040. In contrast, developed countries are expected to experience a 24% increase in ALS cases. A subsequent investigation by Gowland et al. [13] forecasts a 30% rise in ALS incidence within the United Kingdom over a similar timeframe.

These projections are largely determined by demographic shifts: an increase in the proportion of the elderly population, a decrease in mortality and fertility rates, and an increase in the availability of diagnostic services. This growth will inevitably lead to an increase in the social and economic burden of the disease and to significant expenditures of the healthcare system.

Thus, the high mortality risk, the rapid progression, the preserved cognitive functions, and the lack of etiotropic therapy make ALS one of the most dramatic forms of neurodegeneration, which necessitates a deeper understanding of the molecular basis of the disease and the development of new treatment approaches.

According to modern views, ALS is a multifactorial disorder, with its risk determined by a complex combination of genetic, epigenetic, and environmental factors. As known to date, more than 50 genes are involved in the disease progression in 50% of patients with familial ALS and 7.5% with sporadic ALS [14]. The most common mutations are the expansion of the GGGGCC hexanucleotide repeat in the chromosome 9 open reading frame 72 (*c9orf72*) gene and the point mutations of Cu/Zn superoxide dismutase 1 (*sod1*) gene leading to toxic aggregations of the SOD1 protein. In rarer cases, mutations occur in the *tardbp* gene on chromosome 1p36 encoding TAR DNA-binding protein 43 (TDP-43), a nuclear RNA-binding protein involved in the regulation of transcription, processing, and splicing of mRNA [15]. However, more than 90% of patients with ALS have hyperphosphorylated, ubiquitinated aggregates of TDP-43 formed through impaired transcription and translation of *tardbp* [16].

Thus, while genetic factors exert a demonstrable influence on ALS pathogenesis, their role is not invariably causative, thereby highlighting the significance of epigenetic mechanisms. Evidence suggests an inverse correlation between DNA methylation and elevated expression levels of mutant TDP-43, positing a relationship with TDP-43 proteinopathy [17]. Furthermore, demethylation of the DNA within the autoregulatory region encoding the 3′-untranslated region of *tardbp* (3′UTR) has been shown to correlate with increased levels of cytoplasmic TDP-43 protein within affected neurons. Histone modifications also contribute to elevated TDP-43 protein levels and potentiate aggregate formation [18]. Moreover, a significant reduction in microRNA abundance is observed in ALS, potentially accounting for the increased expression of specific ALS-related transcripts and proteins, and the subsequent formation of their aggregates.

These findings underscore the intricate nature of ALS pathogenesis, wherein genetic and epigenetic determinants are inextricably linked and subject to modulation by environmental factors influences. Among the major environmental factors that have recently received increasing attention are head and spine injuries, exposure to heavy metals, pesticides, high-intensity radar waves, and transmitted infectious diseases caused by enteroviruses, retroviruses, and polioviruses [19,20,21]. Their respective contributions or mechanisms remain largely unknown. One of the hypotheses is that ALS may be caused by certain environmental factors in combination with genetic predispositions [22].

**Figure 1:**
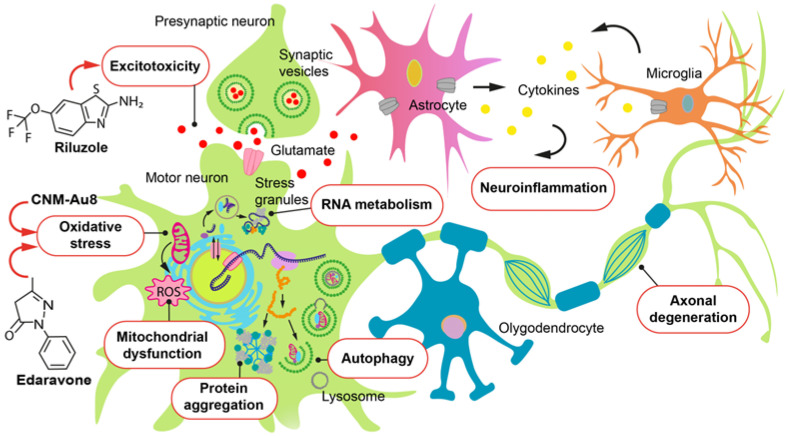
Major pathogenesis mechanisms involved in progression of amyotrophic lateral sclerosis (ALS). The pathophysiological mechanisms driving neurodegeneration in ALS are multifaceted and arise from a complex interplay of molecular and genetic pathways. The key processes implicated include the enhanced production of reactive oxygen species, glutamate-mediated excitotoxicity, mitochondrial dysfunction, impaired axonal transport, and accumulation of cytoplasmic protein aggregates such as SOD1, TDP-43, and fused in sarcoma (FUS). Furthermore, the activation of astrocytes and microglia leads to the release of pro-inflammatory cytokines, which contributes to neuroinflammation and subsequent degeneration of motor neurons.

Ultimately, these etiological factors converge on the defining neuropathology of ALS: the selective degeneration of motor neurons within the motor cortex, brainstem, and anterior horns of the spinal cord. It occurs through the activation of complex pathophysiological mechanisms (Figure 1) including excitotoxicity [23], oxidative stress [24], mitochondrial dysfunction [25], formation of protein aggregates [26], dysregulation of autophagy processes [27], neuroinflammation [28], and disruption of post-transcriptional modification of RNA and axonal transport [29].

## 2. Glutamate Excitotoxicity and Antiglutamatergic Drugs

Glutamate-mediated excitotoxicity is considered a key mechanism in the pathogenesis of ALS [30,31]. This hypothesis is supported by the observation of elevated extracellular glutamate levels in both the blood and cerebrospinal fluid (CSF) of *sod1^G93A^* mutant mice and patients with ALS [32,33,34]. Glutamate is the primary excitatory neurotransmitter in the central nervous system (CNS).

At the molecular level, glutamate is primarily released from presynaptic neurons and activates ionotropic receptors on the postsynaptic membrane. These include N-methyl-D-aspartate (NMDA) receptors, calcium-permeable α-amino-3-hydroxy-5-methyl-4-isoxazolepropionic acid (AMPA) receptors lacking the GluR2 subunit, and kainate receptors. Activation of these receptors leads to sodium (Na^+^) and calcium (Ca^2+^) influx, postsynaptic depolarization, and generation of an action potential required for synaptic transmission.

**Figure 2:**
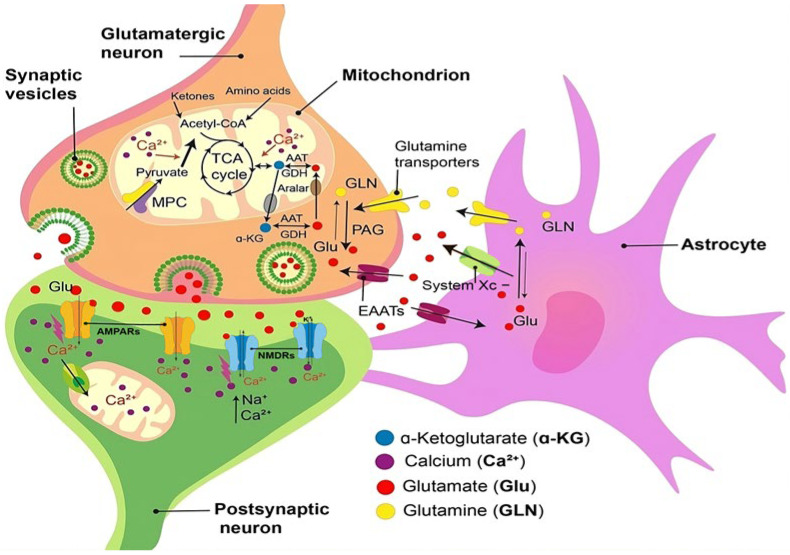
Mechanisms of development of glutamate-mediated excitotoxicity. Excessive glutamate release occurs both at presynaptic terminals and from astrocytes via the cystine/glutamate antiporter (system Xc^−^). Concurrently, impaired clearance of extracellular glutamate, due to dysfunction of EAATs, exacerbates its accumulation within the synaptic cleft. This pathological elevation in synaptic glutamate leads to sustained overactivation of ionotropic glutamate receptors, particularly NMDA and AMPA. The ensuing Ca^2+^ influx into postsynaptic neurons initiates a cascade of intracellular disturbances. Calcium overload disrupts cellular homeostasis by activating proteolytic enzymes, amplifying the generation of ROS, and inducing mitochondrial dysfunction. Collectively, these processes impair neuronal bioenergetics and promote cell death through both acute necrotic mechanisms and delayed apoptotic pathways.

In ALS, glutamate homeostasis is disrupted. This is likely due to both increased glutamate release and impaired clearance within the CNS. On one hand, excessive glutamate release from glutamatergic presynaptic terminals and, to a lesser extent, from astrocytes via the cystine/glutamate antiporter (system xc^−^) (Figure 2) has been demonstrated in spinal cord slices from transgenic *sod1^G93A^* mice. Additionally, dysregulation of calcium-permeable AMPA receptors contributes to excitotoxicity [35].

On the other hand, insufficient reuptake of glutamate from the synaptic cleft further exacerbates its accumulation. This process is primarily mediated by excitatory amino acid transporters (EAAT 1–5), which are predominantly expressed on perisynaptic astrocytic processes and, to a lesser extent, on neuronal dendrites. EAAT2 and glutamate transporter 1 (GLT-1) is the main glutamate transporter in the brain, responsible for clearing approximately 90% of extracellular glutamate, except in regions such as the cerebellum and retina, where EAAT1 is more prevalent. Notably, antisense oligonucleotide-mediated suppression of EAAT2/GLT-1 in adult rats has been shown to induce progressive motor neuron degeneration [36,37].

Persistent overactivation of glutamate receptors results in a pathological influx of Ca^2+^, followed by calcium release from intracellular stores. This cascade leads to mitochondrial membrane depolarization and dysfunction of mitochondrial transporters and respiratory chain enzymes. Consequently, oxidative phosphorylation is impaired, and levels of reactive oxygen (ROS) and nitrogen species (RNS) increase, triggering oxidative stress [38,39].

Oxidative stress activates several intracellular enzyme systems. These include calcium-dependent proteases that degrade cytoskeletal proteins and membrane-associated enzymes, as well as phospholipase A2, which hydrolyzes membrane phospholipids and releases arachidonic acid—a precursor of pro-inflammatory prostaglandins and lipid peroxidation. Furthermore, nuclear endonucleases are activated, leading to DNA fragmentation and promoting both apoptotic and necroptotic cell death.

Importantly, glutamate excitotoxicity also drives neuroinflammatory processes (Figure 1). Damaged neurons and glial cells release danger-associated molecular patterns (DAMPs), which activate microglia via Toll-like receptors (TLRs) and the NLRP3 inflammasome [40]. This results in the production of pro-inflammatory cytokines such as interleukin-1β (IL-1β) and tumor necrosis factor-alpha (TNF-α), along with further glutamate release [41].

This vicious cycle characterized by impaired glutamate clearance, calcium overload, mitochondrial dysfunction, oxidative stress, and neuroinflammation constitutes the core of glutamate-mediated excitotoxicity in ALS. It underscores the relevance of this mechanism as a promising therapeutic target in the treatment of ALS.

**Figure 3:**
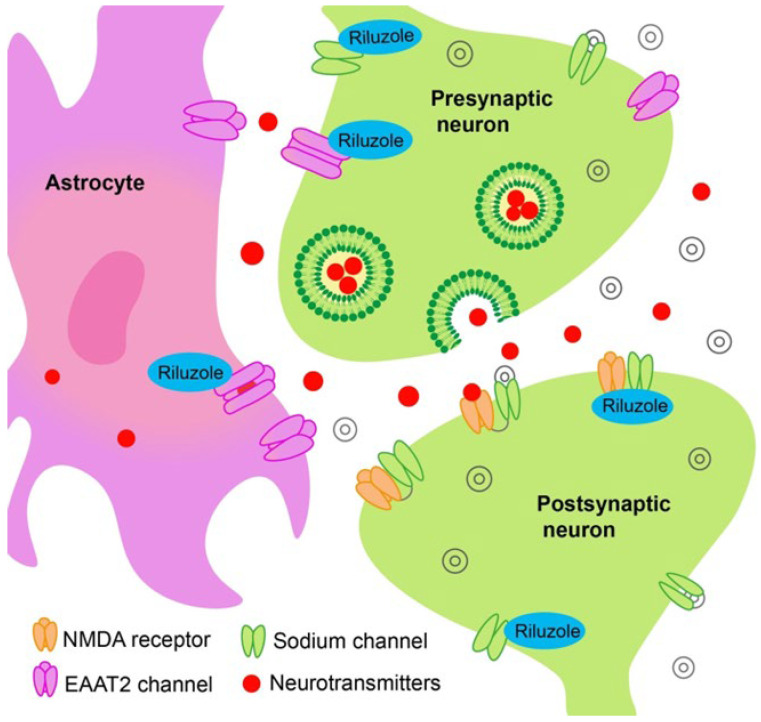
Mechanisms of riluzole action: it exerts a modulatory effect on Na^+^- and late K^+^-dependent ionic currents in neurons, leading to a reduction in glutamatergic transmission at the postsynaptic membrane. It also inhibits the activity of NMDA receptors and decreases the release of glutamate from presynaptic neurons. Furthermore, riluzole promotes the uptake of extracellular glutamate by astrocytes and inhibits protein kinase, which is a key enzyme in the process of hyperphosphorylation and aggregation of the TDP-43 protein. Additionally, by acting on astrocytes, riluzole increases the expression of EAAT2, which is involved in the uptake of glutamate from the synaptic cleft.

The first drug approved by the U.S. Food and Drug Administration (FDA) in 1995 was riluzole (2-amino-6-(trifluoromethoxy)benzothiazole), a benzothiazole derivative that blocks glutamatergic neurotransmission in the CNS mainly at the presynaptic level and, presumably, has neuroprotective, antioxidant, and antiapoptotic effects [42,43,44]. The molecular mechanisms of action of riluzole are diverse, very complex, and not fully understood. As known (Figure 3), riluzole is dose-dependently involved in the blockage of voltage-dependent sodium and calcium channels, thereby reducing the flux of Na^+^ and Ca^2+^ through the postsynaptic membrane [45,46,47], non-competitively inhibits NMDA receptors [48], reduces the release of glutamate from presynaptic terminals [49,50], and enhances the astrocytic uptake of extracellular glutamate [51,52]. Furthermore, it inhibits catalytic activity of casein kinase 1δ (CK 1δ), the main enzyme involved in hyperphosphorylation and aggregation of TDP-43 in cell models in vivo and in vitro, thereby preventing the cascade of events with TDP-43 compartmentalization at the cytoplasmic level and maintaining the TDP-43 homeostasis and normal expression of the EAAT2 transporter by astrocytes [53,54,55]. The above finding suggests an indirect effect of riluzole on two key links in the ALS pathogenesis: glutamate excitotoxicity and proteinopathy. No evidence of a direct effect of riluzole on the TDP-43 protein aggregation has been obtained to date [56].

The therapeutic efficacy of riluzole was evaluated in four large-scale randomized controlled trials (RCTs), of which the earliest was conducted in 1994 by Bensimon with co-authors [57]. The trial involved 155 outpatients under 75 years with the onset of their first ALS symptoms less than five years before and with a forced vital capacity of more than 60%. All patients were stratified according to the site of onset of disease: bulbar-onset or limb-onset disease. During the study, one group of patients received riluzole at a daily dose of 100 mg, while the other received placebo [57,58]. The primary efficacy outcomes were defined as tracheostomy, death, or a change in functional status after 12 months of treatment. The results demonstrated a statistically significant difference in the survival rate of riluzole vs. placebo patients, with a median survival of 449 days in the placebo group vs. 532 days in the riluzole group. The deterioration of muscle strength was significantly slower in the riluzole group compared to the placebo group (*p* = 0.028). For patients with bulbar-onset disease, the one-year survival rate was 35% in the placebo group and 73% in the riluzole group (*p* = 0.014), whereas for patients with limb-onset disease, one-year survival was 64 and 74%, respectively (*p* = 0.17). In general, the riluzole therapy reduced mortality by 38.6% at 12 months and by 19.4% at 21 months.

As a confirmation of the success of the first riluzole study and in order to determine its dose-dependent effect, Lacomblez et al. (1996) [58] conducted the second trial that involved 959 patients with clinically definite or probable ALS according to the El Escorial World Federation of Neurology criteria. Similarly to the first study, the patients were divided into groups depending on the site of ALS onset. The outcomes were survival without a tracheostomy and rate of change in functional measures (muscle strength, stiffness, and respiratory function). An analysis of the results showed a dose-dependent increase in the survival of riluzole-treated ALS patients vs. placebo patients after 18 months. Similarly to the first study, riluzole increased the survival without tracheostomy and possibly decreased the rate of muscle-strength loss. However, it did not show a difference in therapeutic effect between the forms of ALS onset. The positive results such as the dose-dependent reduction in the risk of death were also accompanied by adverse reactions in the form of increased liver enzyme activities, which were more common at a riluzole dose of 200 mg. Taking into account the efficacy and safety results, an optimal dose of riluzole at 100 mg/day had the best benefit-to-risk ratio [58].

In parallel with this study, the effect of riluzole was determined for ALS patients older than 75 years or those with advanced stage of disease. The study included 168 patients over 75 years, randomized to either riluzole or placebo, that were monitored for 8 months. Riluzole was well tolerated by both groups of patients, and the observed adverse events were similar in nature and frequency to those previously reported. The study did not reveal any statistically significant difference in survival between the riluzole group and the placebo group, probably due to a too-small sample size [59].

In subsequent studies, ambiguous results were obtained. Most riluzole trials reported its positive effect on increase in survival of ALS patients vs. placebo patients [60,61,62,63,64,65,66,67]. Mitchell with co-authors [63], using the Cox model with both forward and backward selection, found that the risk of death was significantly reduced in riluzole-treated patients vs. untreated patients (*p* < 0.001). Similarly, Stevic with co-authors (2016) [65] carried out a multivariate regression analysis and found that riluzole treatment had a positive effect on survival of patients. However, the reported increase in the median survival of more than 18 months, with an average of 6–19 months, was significantly longer compared to previous results where survival had increased by an average of 3–5 months [67]. It is noteworthy that the increase in survival of riluzole-treated ALS patients occurred, to a greater extent, due to the longer time in clinical stage 4 and, to a lesser extent, due to an increase in the time in stages 1–2, which suggests at least two mechanisms of action: early, through neuroprotection, and late, which has an effect on mainly the respiratory function [68,69,70,71].

In a smaller number of studies, a clinically significant effect of treatment was reported (with an increase in the median survival of ≥3 months), but there was no statistically significant difference compared to the placebo group [72,73,74]. Although Chen et al. (2016) [74] reported the lack of statistically significant difference between the groups in their study, they, nevertheless, showed that long-term use of riluzole was associated with a better prognosis for ALS patients who were administered 100 mg/day riluzole compared to non-riluzole-treated patients, which indicates the efficacy and feasibility of long-term administration of riluzole to increase patients’ survival.

Nevertheless, studies did not find any significant effect of treatment between the riluzole group and the placebo group (<3 months) [75,76].

In all studies, patients showed high adherence to treatment and good tolerability of the drug, as evidenced by the lack of a statistically significant difference in the frequency of side-effects between the trial group and the placebo group. Most of the side-effects were minor and reversible. The most frequently reported side-effects were increased fatigue, headache, gastrointestinal syndrome (nausea, decreased appetite, abdominal discomfort, and diarrhea) and an increase in liver enzymes, which had a direct relationship with the dose received and an inverse relationship with the duration of administration [57,58,77,78]. At the recommended dose of riluzole of 100 mg/day, the rate of marked elevation of the alanine aminotransferase (ALT) level more than five-fold from the reference values was 3.8%, which is less than half of the rate of moderate elevation of ALT levels (from three- to five-fold vs. the reference values), 10.6%, which emphasizes the importance of monitoring the level of liver enzymes in riluzole therapy. Neutropenia, a rare but potentially serious side-effect, was described by Wagner et al., 1997 and Weber et al., 2004 [79,80] in studies on three patients that received long-term treatment with riluzole. Another rare side-effect was interstitial pneumonia, described in a study by Yanagisawa et al. [81]. The clinical picture included an unproductive cough and shortness of breath. After discontinuation of the drug, the symptoms regressed. A number of studies indicate probability of acute pancreatitis to develop against the background of riluzole therapy, e.g., Ianiro et al., 2014 [82], with four confirmed cases where symptoms included epigastric pain, increased amylase, and lipase [82,83,84,85]. All cases required drug discontinuation.

In recent years, the effect of riluzole on the functional state of ALS patients was also reported. Brooks with co-authors [86] studied muscle strength in 128 ALS patients before the start of riluzole therapy and after four weeks using the Medical Research Council (MRC) scale. By the end of four weeks, 62.5% of patients showed improvement in one or more of the muscles most commonly involved in ALS. However, the evidence is still insufficient for any firm conclusions, and more extensive research is required. Thus, the more than 25-year clinical trials of riluzole confirm its efficacy and safety in the ALS treatment.

Riluzole is available in the form of film-coated tablets, 50 mg. The recommended dose is 50 mg twice a day. The therapy should be started as early as possible since the ALS diagnosis and continue until the end of life. However, with the progression of the disease and the onset of dysphagia, which occurs in about 50% of ALS patients at the onset of the disease (in 94.7% with bulbar-onset ALS and in 35.2% of patients with other forms of disease onset) and in more than 80% at late stages, taking tablets of the drug is associated with significant difficulties eventually leading to a lower level of adherence to treatment and self-termination of therapy [87,88]. In recent years, bioequivalent, alternative forms of the drug have been developed: a suspension (under the trade name Teglutik) for oral administration and a sublingual disintegrating film (under the trade name Exservan) that dissolves on the tongue without swallowing movements, which is especially important in the case of progressive dysphagia when taking tablets is difficult [89].

### Other Drugs with Effect on Glutamate-Mediated Neurotransmission

Ionotropic AMPA receptors, providing the glutamate-mediated postsynaptic entry of Ca^2+^ ions and the activation of Ca^2+^-dependent signaling systems, have been proposed as therapeutic targets that reduce pathological glutamate stimulation. The drug named talampanel, a non-competitive antagonist of AMPA receptors, showed a moderate reduction in the rate of ALS progression on the revised ALS functional rating scale (ALSFRS-R) but did not have a significant effect on patients’ survival [90]. Perampanel, another drug that is also an AMPA receptor antagonist, in the first phase of a preclinical trial demonstrated a statistically significant slowdown in ALS progression and a decrease in motor neuron death in AR2 and AR2H mice [91]. However, in a clinical trial, it caused pronounced side-effects such as aggressiveness, daytime drowsiness, and an increase in dysarthria, while having no significant effect on the muscle strength and mobility of ALS patients on the ALSFRS-R [92]. Another approach to removing excess glutamate from the synaptic cleft was to increase the expression of the glutamate transporter EEAT2 using ceftriaxone, which, however, has not yielded any successful clinical results either [93]. All the above findings emphasize the need for a more detailed study of glutamate-mediated toxicity in the ALS pathogenesis.

## 3. Oxidative Stress and Antioxidant Drugs

Due to the high intensity of oxidative metabolism in brain cells and the high level of polyunsaturated fatty acids in them, especially docosahexaenoic acid, oxidative stress plays a crucial role in the ALS pathogenesis. The oxidative stress results from an imbalance between the excessive production of reactive oxygen species (ROS) and the insufficient compensatory capabilities of antioxidant systems [94]. ROS are radical and non-radical forms of oxygen such as superoxide anion radical (O_2_^−^), hydroxyl radical (OH^−^), hydrogen peroxide (H_2_O_2_), and singlet oxygen formed as byproducts of cellular metabolism through enzymatic and non-enzymatic reactions with partial reduction of oxygen. In motor neurons, most intracellular free radicals are generated by the mitochondrial respiratory chain through the synthesis of adenosine triphosphate (ATP) molecules and oxidative phosphorylation [95,96]. After being formed, ROS damage proteins, lipids, and nucleic acids and initiate production of reactive nitrogen (RNS) and sulfur species (RSS), which leads to disruption of cellular metabolism, activation of lipid peroxidation, initiation of neuroinflammation, and, eventually, to the death of neurons [97]. The removal of excess ROS by the antioxidant system, represented by superoxide dismutase (SOD), catalase, glutathione peroxidase, glutathione reductase, vitamins A, C, and E, and glutathione, becomes inefficient because of the decrease in its capacities under a sharp ROS increase. Furthermore, there is evidence of impaired functioning of certain antioxidant system components in patients with ALS. A decreased activity of the antioxidant properties of glucose-6-phosphate dehydrogenase in red blood cells was observed in patients with sporadic ALS [98]. A pathology was also found in the regulation of glutathione homeostasis in the cytoprotective system of the antioxidant response element (ARE) associated with nuclear E2-related factor 2, Nrf2 [99,100,101]. A mutation in the *sod1* gene was studied that leads to the loss of functions of the SOD enzyme catalyzing the conversion of superoxide radical into hydrogen peroxide and deteriorates the oxidative damage through the activation of prooxidant pathways. A direct stimulating effect of the product of the mutant *sod1* gene on NADPH oxidase in transgenic mice and human cell lines was discovered.

This oxidative damage to neurons causes an increase in oxidative stress markers and lipid peroxidation products such as 8-hydroxy-2′-deoxyguanosine, 4-hydroxynonenal, and ascorbate free radicals in the motor cortex of the brain, in the spinal cord, and in glial cells [102,103]. An increase in their concentration was observed in cerebrospinal fluid (CSF) and in the blood of ALS patients, which was used as a basis for the development of antioxidant drugs as potential therapeutic agents [104].

## 4. Edaravone

Edaravone became the second drug approved for ALS treatment by the FDA. At first, this drug was actively used in Japan to reduce oxidative stress in acute ischemic stroke [105,106]. As the knowledge of the oxidative stress’ role in the pathogenesis of neurodegenerative diseases extended, edaravone has been approved as a new drug for ALS therapy. The mechanism of its action is still unclear, but in vitro and in vivo studies have shown its antioxidant properties based on the ability to accept free radical electrons and decrease the production of ROS and peroxynitrite both in a hydrophilic medium such as the cytoplasm and in a lipophilic medium, the cell membrane, thereby exerting neuroprotective and anti-inflammatory effects through oxidative stress reduction [107].

A study of the safety and efficacy of edaravone was conducted for 24 weeks in a Phase III trial (NCT01492686) in a cohort of Japanese patients with confirmed or probable ALS according to El Escorial criteria and with a forced vital capacity (FVC) of more than 80%. All patients involved in the study had the opportunity to continue the previously initiated riluzole therapy. The results of the trial showed a 33% slowdown in disease progression compared to the placebo group, a reduction in the rate of decline in the ALSFRS-R score (−5.01 vs. −7.50 in the placebo group, *p* = 0.0013), and a significant improvement in the quality of life score on the forty item ALS assessment questionnaire (ALSAQ-40) scale in patients that received intravenous edaravone [108]. According to the study, 84% of edaravone-treated patients reported side-effects, the most common of which were contusions at the injection site (19% vs. 13% in the placebo group), contact dermatitis (12% vs. 4%), and constipation (12% vs. 12%). Serious side-effects, observed in 16% of patients that received edaravone, included severe dysphagia (12% vs. 12% in the placebo group), respiratory dysfunction (3% vs. 3%), and speech disorders (1% vs. 3%) [108].

Subsequent studies to assess the efficacy of edaravone in larger samples of ALS patients did not show its positive effect on the rate of disease progression. Edaravone was well tolerated by patients, but the most common side-effects were also bruises after injections (15%), gait disturbance (13%), and headache (10%) [109,110].

A study of the effect of edaravone on survival was conducted in a retrospective comparative analysis on patients with ALS treated with intravenous edaravone in the period from 2017 to 2020. The pre-index disease duration for the treatment and control groups at the study start was approximately 7 months, and both groups were comparable in the proportion of patients that had a history of riluzole prescription (65.4% vs. 65.4%). The median duration of edaravone treatment was 8.6 months. The trial results demonstrated an increase in median survival (29.5 months for the edaravone-treated cases vs. 23.5 months for non-edaravone-treated controls) and a 27% lower risk of death for edaravone-treated cases than for non-treated controls (*p* = 0.005) [111]. The results obtained differed from previously known data, as reported in a study by Vu M. et al., 2020 [112] that showed a slight decrease in death rates (per 100 patient-years) with edaravone treatment (18.0) compared to treatment with riluzole only (29.3). Such differences can probably be related to genetics, since there are ethnic differences in the efficacy of the drug between different groups of patients [113], as evidenced by the positive research results in Japan [105] and the United States [111] but not in Germany [110] and Italy [109].

A meta-analysis of recent trials confirmed the efficacy of edaravone in improving the survival of patients with ALS after 18, 24, and 30 months compared to the control group and showed a relationship of edaravone treatment with a lower incidence of musculoskeletal disorders [114]. Thus, edaravone may be considered as one of the effective components of ALS therapy.

The last analysis of edaravone side-effects, conducted from 2017 to 2024 and containing results of 2986 reports, which has been published in 2025, showed that the most common of them were asthenia (*n* = 135), gait disorders (*n* = 99), respiratory failure (*n* = 40), and respiratory disorders (*n* = 29). The most significant side-effects were thrombosis at the injection site, gastric fistula, edema at the injection site, and infections associated with an intravenous catheter [115].

Due to the high frequency of side-effects associated with intravenous administration of the drug, an oral form of edaravone, aimed to facilitate drug delivery, was approved in the United States in May 2022 (FAB122, Radicava ors). The Phase I trial showed the bioequivalence of the suspension for oral administration at a dose of 105 mg [116].

In ADORE Phase III clinical trial (NCT0178810), conducted in several major European centers, the drug administered at a dose of 100 mg, in addition to the main ALS therapy, was compared to placebo for 48 weeks. According to the results, the oral suspension of edaravone did not show significant benefits compared to placebo in slowing down the disease progression after 48 weeks of daily intake, which was measured as variations in the ALSFRS-R scores. Also, researchers did not observe any improvement compared to placebo in long-term survival, measured on the Combined Assessment Function and Survival (CAFS) scale, at 48 and 72 weeks [117]. The results may presumably be explained by the drug formula different from the original drug, as stated by Mitsubishi Tanabe Pharma America. The failure of Phase III resulted in suspending further study on the oral form of edaravone, and it was decided to discontinue the larger-scale open-label extension (ADOREXT) study (NCT05866926). However, the need for an effective oral form of edaravone still exists, as it will allow avoiding the complications of intravenous administration.

## 5. Gold Nanocrystals, the Drug CNM-Au8

Disturbances of metal metabolism, mainly of copper, iron, and zinc, are also reported as a common cause of neuronal oxidative stress. This finding has become a basis to study CNM-Au8, an aqueous suspension of gold nanocrystals with a catalytic ability to increase the efficiency of key metabolic reactions while reducing the ROS level. At the molecular level, CNM-Au8 catalyzes the oxidation of nicotinamide adenine dinucleotide (NADH·H^+^) to NAD^+^, which is a co-factor in redox reactions associated with ATP synthesis in the respiratory chain of mitochondria [118].

The potential of CNM-Au8 as a drug for ALS treatment has been studied using several in vitro and in vivo models [118]. It was found that the effect of CNM-Au8 in mouse and cell culture models is related to a dose-dependent increase in survival of motor neurons and, vice versa, to a decrease both in the markers of oxidative stress and in the TDP-43 aggregation in lower motor neurons of rodents. Furthermore, CNM-Au8 prolonged the survival of upper motor neurons with a mutation in the *c9orf72* gene and led to a dose-dependent increase in survival and motor function in a model of transgenic mice with ALS (*sod1^G93A^*). No side-effects associated with the drug were found in the studies, which may indicate its potential safety. The success of the preclinical studies suggested this drug as a possible therapeutic strategy for ALS treatment and also laid the foundation for the start of clinical trials.

In RESCUL-ALS Phase II clinical trial (NCT04098406), launched in 2019, the clinical effect of the drug was determined in patients with the initial stage of ALS who received 30 mg of CNM-Au8 daily over 36 weeks [119]. The results were analyzed using a neurophysiological biomarker such as summed motor unit number index (MUNIX) and indicators of respiratory function such as the ALSFRS-R score change and change in quality of life (ALSSQOL-SF, ALS-Specific Quality of Life instrument and its revised version). At the end of the study, most of the participants (90%) proceeded to an open multicenter study (NCT05299658) where all patients from both groups continued to receive CNM-Au8 for 120 weeks. As a result of these studies, no statistically significant difference in neurophysiological parameters was observed between the treatment group and the placebo group. However, there was an absolute reduction in the risk of disease progression by 55%, measured from the time of diagnosis to the death, initiation of non-invasive ventilatory support, or gastrostomy tube placement (*p* = 0.0125). Also, in the group of patients administered CNM-Au8, there was a slower progression of the disease, which was manifested as a reduction in the proportion of patients free of >6-point ALSFRS-R decline (*p* = 0.035). An improvement in quality of life was also registered on the ALSSQOL-SF scale (*p* = 0.0177). The potential clinical benefits, confirmed in the RESCUL-ALS study, were accompanied by a significant increase in survival also in open label extension (OLE), as evidenced by a 60% reduction in all-cause mortality in the cohort with CNM-Au8 treatment (*p* = 0.0429) [119]. These clinical effects were accompanied by a marked decrease in the level of neurofilament-light (NF-L) chains in blood serum, which may likely be a consequence of the neuroprotective effect of CNM-Au8. It is noteworthy that the drug had a more pronounced effect on patients with predominantly peripheral symptoms. CNM-Au8 was well tolerated, which allows considering it a potentially effective drug for ALS treatment and including it in a larger multicenter study of the HEALEY ALS platform (NCT04297683) that was launched in 2021 and is scheduled to end in 2025 [120].

## 6. Verdiperstat

Attempts to expand the range of therapeutic opportunities for treating ALS have led to the study of the drug verdiperstat, an irreversible inhibitor of myeloperoxidase. According to the first results of the Phase II/III clinical trial (NCT04297683), which started in 2021, there were no statistically significant differences between the verdiperstat group and the placebo group both in survival and in the ALSFRS-R score change at the study start and after 24 weeks [121]. Also, the drug neither had any effect on muscle strength nor did it extend survival before the start of respiratory support. Eventually, this drug was found to be ineffective in ALS. It is likely that the results obtained, as in the case of TCH346, may be associated with the short follow-up periods for patients, which did not allow researchers to observe the clinical effect of the treatment [122]. Thus, this drug requires a further, longer-term study.

## 7. Mitochondrial Dysfunction and Cytoprotective and Antiapoptotic Drugs AMX0035

AMX0035 (under the trade name Relyvrio) is the first combined, potentially disease-modifying drug approved in 2022 by the FDA for ALS treatment. It is a patented, fixed combination of sodium phenylbutyrate and taurursodiol (tauroursodeoxycholic acid) to be administered orally. This drug was developed by Amylyx Pharmaceuticals as a therapeutic approach targeted at several pathophysiological mechanisms of ALS [123].

Taurursodiol, being a hydrophilic secondary bile acid synthesized mainly in the liver, has a cytoprotective effect due to the chaperone activity that relieves the severity of endoplasmic reticulum stress and provides adequate protein folding and maturation, as evidenced by preclinical studies on mouse models and cell cultures [124,125,126]. The antiapoptotic effect of taurursodiol is exerted through the reduction in translocation of the proapoptotic Bcl-2-associated X (BAX) protein from cytoplasm to mitochondria, stabilization of the mitochondrial membrane, modulation of mitochondrial activity, suppression of mitophagy, and increase in the apoptosis threshold [126,127,128,129]. A decrease in the cyclin D1 expression by taurursodiol also presumably influences the cell cycle control [130].

A pilot study, completed in 2015, evaluated the safety and efficacy of taurursodiol for the ALS treatment [131]. The trial involved 34 patients with ALS administered the drug AMX0035 orally at a daily dose of 2 g in combination with riluzole. By the end of the study, the authors recorded not only the safety of this drug but also the positive effect of slower ALS progression in the treatment group vs. the placebo group (*p* < 0.01) and also higher ALSFRS-R scores in the trial group vs. the placebo group (*p* = 0.007).

In view of the positive results of the pilot trial, the study of taurursodiol was continued with a larger sample of 172 patients with diagnosed ALS according to Revised El Escorial criteria [132]. The treated patients were divided into subgroups on the basis of taurursodiol dosage (≤1000 or >1000 mg/day) and the duration of treatment (≤12 or >12 months). Among the results of the study, there was a dose-dependent increase in the median overall survival of taurursodiol-treated patients to 49.6 vs. 36.2 months in the control group. The drug was well-tolerated throughout the study; side-effects were observed in 20.9%, of which diarrhea (14.0%), abdominal pain (5.8%), and skin eruption (3.5%) were the most common.

The second component of the fixed combination of AMX0035, sodium phenylbutyrate, is a short-chain fatty acid that penetrates the blood–brain barrier (BBB) and, like taurursodiol, acts as a pharmacological chaperone through the interaction of the hydrophobic region with open hydrophobic parts of unfolded proteins, thereby reducing their aggregation [133]. Thus, the fixed combination of the drugs likely has an effect on both mitochondrial dysfunction and proteinopathy developing in ALS.

In a study on a model of ALS transgenic mice expressing the mutant forms of SOD1 (G93A H1 high-expressor strain), sodium phenylbutyrate showed good efficacy by inhibiting programmed cell death and ameliorating disease progression [134]. In particular, it was found to affect the phosphorylation of the IkB inhibitor, which led to translocation of the nuclear factor-kB (NF-kB) p50 into the cell nucleus and subsequent transactivation of *bcl-2* gene expression. The *bcl-2* gene, encoding the beta cell lymphoma 2 (bcl-2) protein, prevented the release of cytochrome *c* from mitochondria and, as a result, inhibited the activation of caspases, thereby slowing down the process of motor neuron death. Thus, by influencing both transcriptional and post-translational mechanisms, sodium phenylbutyrate promoted the motor neuron survival and reduced the rate of disease progression in pre-clinical models of ALS. These findings became the basis for proposing sodium phenylbutyrate as a promising therapeutic agent for ALS treatment. The assumption that a fixed combination of several drugs acting on various pathogenetic links of ALS can provide a more pronounced therapeutic effect was a key incentive for further clinical trials. The efficacy and safety of the combination therapy based on sodium phenylbutyrate and taurursodiol was studied in the CENTAUR Phase II clinical trial of AMX0035 (NCT03127514), which involved 137 patients with definite or probable ALS diagnosed according to El Escorial criteria, within the previous 18 months. All participants were randomly assigned in a 2:1 ratio to receive AMX0035 (3 g of sodium phenylbutyrate and 1 g of taurursodiol) or placebo for 6 months. Patients who completed the 6-month randomized trial had the opportunity to continue taking phenylbutyrate and taurursodiol in an open-label extension trial for up to 132 weeks. Patients were evaluated at baseline and every 3 weeks for a 24-week period. Upon completion of the trial, a slowdown in the mean rate of functional activity decline in the ALSFRS-R score was −1.24 points per month in patients that actively received AMX0035 vs. −1.66 points per month in placebo patients. Secondary outcomes did not differ significantly between the two groups [135]. The median overall survival was 25.0 months in the treatment group and 18.5 in the placebo group [136]. Thus, the treatment with AMX0035 resulted in an increase in median overall survival by 6.5 months vs. the placebo group. The risk of any key event was 47% lower in those patients who were initially randomized to the drug compared to placebo (*p* = 0.003). The risk of death or use of permanent breathing support (including tracheostomy) was 49% lower among those who had initially received the drug compared to placebo (*p* = 0.007). The risk of hospitalization was 44% lower in the treatment group vs. the placebo group (*p* = 0.03).

In a 48-week PHOENIX Phase III trial (NCT05021536), the safety and efficacy of AMX0035 was determined in 664 ALS patients divided into a group that received AMX0035 and a placebo group. During the study, patients were allowed to continue the previously initiated therapy with riluzole, edaravone, or both. The efficacy of AMX0035 was judged by the change in the ALSFRS-R score compared to the baseline level, with the secondary endpoints including evaluation of quality of life, overall survival, and external respiration function. According to the results in March 2024, AMX0035 did not reach the preset primary or secondary endpoints. There was no statistically significant difference in the change in the ALSFRS-R score compared to the baseline level (*p* = 0.667) between the AMX0035 group and the placebo group.

Thus, the clinical data obtained were partially consistent with preclinical expectations, which highlights the gap between preclinical and clinical efficacy of the drug. On the one hand, it may be due to factors such as limited sample size, heterogeneity of the clinical population. On the other hand, the potential differences in bioavailability and pharmacokinetics of the drug may have an impact on human compared to animal models.

Despite the failure of Phase III, further study of one of the AMX0035 components, taurursodiol, was continued in a TUDCA-ALS randomized Phase III trial as a treatment supplementary to riluzole (NCT03800524). The results have not been published to date.

## 8. Conclusions

ALS is a progressive and fatal neurodegenerative disorder characterized by a highly complex and multifactorial pathogenesis. Key mechanisms implicated in ALS include glutamate excitotoxicity, oxidative stress, mitochondrial dysfunction, impaired autophagy, defective axonal transport, and prominent neuroinflammation. This pathophysiological heterogeneity presents a major challenge for the development of effective treatments and necessitates a multi-targeted therapeutic approach.

To date, only three drugs—riluzole, edaravone, and tofersen—have been approved for the treatment of ALS. However, their clinical efficacy remains limited. Riluzole and edaravone provide only modest survival benefits without significant improvement in functional outcomes. Tofersen, on the other hand, is applicable exclusively to patients with mutations in the *sod1* gene, which is relatively rare. These limitations highlight the urgent need for novel therapeutic strategies aimed at more precise modulation of disease-specific mechanisms.

Contemporary research increasingly focuses on the therapeutic potential of cell-based interventions. Various types of stem cells are under investigation, including embryonic stem cells (ESCs), induced pluripotent stem cells (iPSCs), mesenchymal stem cells (MSCs), mononuclear cells (MCs), neural precursor cells (NSCs), and glial progenitor cells (GPCs) [137]. Although most studies are still in early phases, initial findings demonstrate promising immunomodulatory, regenerative, and neuroprotective effects of these cell types in both preclinical and clinical settings [137]. This suggests that stem cell therapies could represent a transformative approach, either as primary treatments or as adjuncts to existing modalities.

In parallel, gene-targeted therapies are rapidly evolving. These include antisense oligonucleotides, RNA interference techniques (such as siRNA and microRNA), and genome editing using CRISPR/Cas9 [138]. Such methods pave the way for personalized treatment strategies, tailored to the genetic and molecular profiles of individual patients. The clinical use of tofersen in SOD1-mutated ALS already exemplifies this targeted approach, encouraging further development of similar therapies for other mutations such as *c9orf72*, *tardbp*, and *fus*.

The identification and application of molecular biomarkers, such as the Janus kinase (JAK)/Signal Transducer and Activator of Transcription (STAT) [139] pathway and mitogen activated protein kinase kinase kinase kinases (MAP4Ks) [140,141], offer additional avenues for intervention. Inhibition of these pathways has shown potential to preserve motor neurons and extend survival in ALS animal models [142]. Moreover, advanced molecular profiling and monitoring techniques, including skin biopsy-based nerve fiber analysis, may enable improved patient stratification, more accurate prognostication, and real-time assessment of treatment efficacy.

Collectively, these developments signify a shift from a one-size-fits-all treatment model to a personalized, multimodal management strategy. This evolving approach integrates pharmacological agents, cell and gene therapies, symptomatic treatment, and multidisciplinary care. Although no current therapy can completely halt or reverse the progression of ALS, ongoing research, expanded clinical trials, and emerging innovations provide a rational basis for hope. Cutting-edge science, combined with individualized treatment frameworks, appears to offer the realistic potential to significantly enhance patient outcomes and alter the trajectory of this devastating disease.

## Data Availability

Data is contained within the article.
